# Supplementary material for: A method for identifying local adaptation in structured populations
Source: PLoS Genet. 2025 Sep 23;21(9):e1011871. doi: 10.1371/journal.pgen.1011871 (PMC12479014; doi:10.1371/journal.pgen.1011871)
Supplement: S1 Text — (PDF) [file pgen.1011871.s001.pdf]

Supporting Information

Comparison of LogAV and Q_{ST}–F_{ST} results

In figure S1 we compare the distribution of p-values between LogAV and Q_{ST}–F_{ST} using the same sampled individuals and measured phenotypes.
