# Supplementary material for: A method for identifying local adaptation in structured populations
Source: PLoS Genet. 2025 Sep 23;21(9):e1011871. doi: 10.1371/journal.pgen.1011871 (PMC12479014; doi:10.1371/journal.pgen.1011871)
Supplement: S2 Text — (PDF) [file pgen.1011871.s002.pdf]

Computation of the p-value associated with the LogAV

Given the posterior distribution of the log-ratio of ancestral variances $P(\text{Log}_{\mathcal{A}V}|D)$ (calling D the total set of data, including information regarding the traits and the co-ancestries), we compute the two-tailed Bayesian p-value associated with the null hypothesis that $\text{Log}_{\mathcal{A}V} = 0$ as:

$$p = 2\min\left(P(\text{Log}_{\mathcal{A}V} \leq 0|D), P(\text{Log}_{\mathcal{A}V} > 0|D)\right), \qquad \text{(S1)}$$

where the factor 2 is a scaling factor arising from accounting for the conditional probability of picking one of the probabilities in the minimum function (which happens to be 0.5 under the null hypothesis). Given the asymptotic property that posterior distributions reflect long-term sampling distribution of the estimators, with sufficiently weakly informative priors [Gelman et al., 2004], such constructed p-values tend to behave like frequentist p-values, and especially, to be uniformly distributed [Shi and Yin, 2021]. This is tightly connected to the asymptotic behavior of credible intervals as confidence intervals under similar assumptions [Gelman et al., 2004]. Although long-term frequentist interpretation of such p-values (and null hypothesis testing framework in general) is not the most natural use of Bayesian statistics [Gelman et al., 2004], we chose to use it here because *(i)* such p-values are easily obtained from a particular model compared to alternatives like information criterium based model selection; *(ii)* it tracks back with habits established in the field to use null hypothesis testing for Q_{ST}–F_{ST} comparison; and most importantly, *(iii)* the biological question tackled here fundamentally requires to test the null hypothesis of neutral evolution.

References

A. Gelman, J. B. Carlin, H. S. Stern, and D. B. Rubin. *Bayesian Data Analysis.* Text in Statisctical Science. Chapman & Hall/CRC Press, Boca Raton, Florida (US), second edition, 2004. ISBN 1-58488-388-X 978-1-58488-388-3.

H. Shi and G. Yin. Reconnecting p-value and posterior probability under one- and two-sided tests. *The American Statistician*, 75(3):265–275, 2021.
