# Supplementary material for: A method for identifying local adaptation in structured populations
Source: PLoS Genet. 2025 Sep 23;21(9):e1011871. doi: 10.1371/journal.pgen.1011871 (PMC12479014; doi:10.1371/journal.pgen.1011871)
Supplement: S3 Text — (PDF) [file pgen.1011871.s003.pdf]

Trait distribution for selection results

We sampled 10 individuals, 5 sires and 5 dams, from the founder populations, and calculated the traits of the F1 individuals resulting from the NCII (North Carolina II) breeding design. All 5 sires mated with all 5 dams yielding 2 offspring per mating pair. Thus, we phenotyped 50 F1 individuals in each of the subpopulations of the metapopulation for all 500 replicate simulations. From these we obtained the mean and standard deviation of the trait values per subpopulation. We show in S2 Fig., the distribution of trait values across subpopulations.
