# Supplementary material for: A method for identifying local adaptation in structured populations
Source: PLoS Genet. 2025 Sep 23;21(9):e1011871. doi: 10.1371/journal.pgen.1011871 (PMC12479014; doi:10.1371/journal.pgen.1011871)
Supplement: S4 Text — (PDF) [file pgen.1011871.s004.pdf]

## $S$-statistics results

### The $S$-statistic and its behavior under neutrality

The $S$-statistic is a measure proposed by [Ovaskainen et al., 2011] to assess deviations from neutral expectations in population-level effects of quantitative traits. It quantifies how likely the observed pattern of population differentiation described by the estimated population-level random effects ($\mathbf{a}^p$) given the expected neutral distribution of population-level effects obtained from random realization of the distribution($\mathbf{a}_R^p$). The neutral expectation is modeled as a multivariate normal distribution with covariance structure dependent on the metapopulation-level coancestry matrix ($\mathbf{\Theta}^p$). Then to compute $S$, we compare the probability density of the observed population-level effects under the neutral expectation to the probability density of random realizations of the neutral model.

$$S = P\left(f_P(\mathbf{a}^p) < f_P(\mathbf{a}_R^p)\right) \tag{S1}$$

An $S$-value close to 1 indicates that the observed pattern is highly unlikely under neutrality, signaling potential selection, whereas $S \simeq 0.5$ suggests a pattern consistent with neutral expectations. $S \simeq 0$, indicates that the observed population effects are more consistent than expected under neutrality. Under neutrality, the distribution of $S$ is symmetric around 0.5 because $f_P(a^P)$ and $f_P(a_R^P)$ are equally likely to be greater or less than each other. This symmetry arises from the inherent properties of the neutral model, where population-level effects follow a multivariate normal distribution centered on the ancestral mean. Without selection, deviations in either direction (larger or smaller likelihoods for $f_P(a^P)$ compared to $f_P(a_R^P)$) occur with equal probability. However, the exact distribution of $S$ under neutrality is not known.

### Asymptotic normality of the posterior mean of $S$

Driftsel Karhunen et al. [2013] estimates $S$ using a Bayesian method that fits the model parameters and generates posterior samples. Since we are using independent replicates of simulated data, each sample provides a different estimate of $S$, and the mean S-statistic is calculated as the average across the posterior samples. Thus the average of a sufficiently large number of independent and identically distributed random variables approaches a normal distribution, regardless of the underlying distribution of individual variables.

# References

M. Karhunen, J. Merilä, T. Leinonen, J. Cano, and O. Ovaskainen. driftsel: an r package for detecting signals of natural selection in quantitative traits. *Molecular Ecology Resources*, 13(4):746–754, 2013.

O. Ovaskainen, M. Karhunen, C. Zheng, J. M. C. Arias, and J. Merilä. A new method to uncover signatures of divergent and stabilizing selection in quantitative traits. *Genetics*, 189(2):621–632, 2011.
