# Supplementary material for: A method for identifying local adaptation in structured populations
Source: PLoS Genet. 2025 Sep 23;21(9):e1011871. doi: 10.1371/journal.pgen.1011871 (PMC12479014; doi:10.1371/journal.pgen.1011871)
Supplement: S5 Text — (PDF) [file pgen.1011871.s005.pdf]

Simulation parameters per scenario

We simulated different demographic and selective scenarios, each run with 500 replicates. All models assumed biallelic loci for both neutral and quantitative trait loci, a mutation rate of $\mu = 10^{-7}$, and freely recombining loci. The trait heritability was $h^2 = 0.8$. Further details of simulation parameters can be found in Table S1.

Simulation Parameters

The selection equation applied in the island and stepping stone models under selection was:

$$W = e^{-\frac{(P - Z_{\text{opt}})^2}{2\omega^2}} \tag{S3}$$

where W is fitness, P is the trait value, Z_{opt} is the optimal trait value (5 for demes 1–4, for Island Model, or 1-10, for Stepping Stone, and -5 for demes 5–8, for Island Model and 11-20 for Stepping Stones.), and $\omega = 10, 22, 50$ represents the peak width of the fitness function, inversely related to the strength of stabilizing selection.
