# Supplementary material for: A method for identifying local adaptation in structured populations
Source: PLoS Genet. 2025 Sep 23;21(9):e1011871. doi: 10.1371/journal.pgen.1011871 (PMC12479014; doi:10.1371/journal.pgen.1011871)
Supplement: S6 Text — (PDF) [file pgen.1011871.s006.pdf]

Quantinemo

We used QuantiNemo's [Neuenschwander et al., 2019] default initialization parameters which leads to maximum initial genetic diversity. For neutral loci, we kept the default "ntrl_ini_allele_model = 0" which initializes the metapopulation with maximum polymorphism, thus the initial frequency at each bi-allelic locus was drawn from a binomial with mean $p = 0.5$. For quantitative trait loci, we also kept the default "quanti_ini_allele_model = 0" where initial allele frequencies are drawn from a discretized normal distribution, with a biallelic marker, it amounts to drawing at random one of the two alleles. Thus frequencies will be also binomial with mean 0.5. All simulations were run with these initial default settings which describe the ancestral population.

References

S. Neuenschwander, F. Michaud, and J. Goudet. Quantinemo 2: a swiss knife to simulate complex demographic and genetic scenarios, forward and backward in time. *Bioinformatics*, 35(5):886–888, 2019.
