# Supplementary material for: A method for identifying local adaptation in structured populations
Source: PLoS Genet. 2025 Sep 23;21(9):e1011871. doi: 10.1371/journal.pgen.1011871 (PMC12479014; doi:10.1371/journal.pgen.1011871)
Supplement: S7 Text — (PDF) [file pgen.1011871.s007.pdf]

Data for calculating coancestries

$\hat{\mathbf{\Theta}}^P$ and $\hat{\mathbf{M}}$ were estimated from the 2000 neutral markers, using the ten parents from each subpopulation for $\hat{\mathbf{\Theta}}^P$ and all phenotyped F_1 individuals for $\hat{\mathbf{M}}$. In practice, it may be difficult to isolate neutral markers from loci causing adaptation although methods exist (e.g [Pouyet et al., 2018]). Including causal loci will probably have little impact however: if the investigated trait is oligogenic, the possibly elevated differentiation at the causal loci will be swamped among the many neutral loci, and if the trait is very polygenic, differentiation at causal loci won't differ from that at neutral ones [Le Corre and Kremer, 2012].

We used the parents of F_1 individuals to estimate $\hat{\mathbf{\Theta}}^P$, but this could be obtained from any random sample of individuals from the parental generation P. In situations where no genotypes of individuals from the parental generation are available, genotypes of some carefully chosen F_1 individuals could be used, insuring they don't come from the same family to avoid the over-representation of some alleles and thus an overestimation of Θ^P. An alternative would be to reconstruct the parental genotypes from the F_1 individuals, providing a sufficient number of individuals per family are available to do this accurately [Jones and Wang, 2010].

The allele-sharing estimator of kinship, as most genomic estimators of kinship (including the standard estimator), will contain negative estimates [Goudet et al., 2018]. We note that it is not the matrices of kinships, but $\mathbf{M}$, that enter variance decomposition, leading to Eq. 8 for the individual component. While $\mathbf{M}$ also contains negative values, we found no instances where it is not positive definite (all eigenvalues were always positive) and thus the matrix can be inverted by standard procedures. As for $\mathbf{\Theta}^P$, we ensure that all its elements are positive by making it relative to the smallest off-diagonal element of the between-population mean allele sharing matrix (Eq. 6).

References

J. Goudet, T. Kay, and B. S. Weir. How to estimate kinship. *Molecular ecology*, 27(20):4121–4135, 2018.

O. R. Jones and J. Wang. Colony: a program for parentage and sibship inference from multilocus genotype data. *Molecular ecology resources*, 10(3):551–555, 2010.

V. Le Corre and A. Kremer. The genetic differentiation at quantitative trait loci under local adaptation. *Molecular ecology*, 21(7):1548–1566, 2012.

F. Pouyet, S. Aeschbacher, A. Thiéry, and L. Excoffier. Background selection and biased gene conversion affect more than 95% of the human genome and bias demographic inferences. *Elife*, 7:e36317, 2018.
