# Supplementary material for: A method for identifying local adaptation in structured populations
Source: PLoS Genet. 2025 Sep 23;21(9):e1011871. doi: 10.1371/journal.pgen.1011871 (PMC12479014; doi:10.1371/journal.pgen.1011871)
Supplement: S8 Text — (PDF) [file pgen.1011871.s008.pdf]

Results compared to theoretical expectation

On S3-5 Fig. we show qq-plots displaying the difference between the theoretical distribution shapes and the observed ones for the different methods. While the S-statistics neutral distribution for the many replicates should follow a normal distribution, the expectation is uniform for the p-value distribution of the other two methods.
