# Supplementary material for: A method for identifying local adaptation in structured populations
Source: PLoS Genet. 2025 Sep 23;21(9):e1011871. doi: 10.1371/journal.pgen.1011871 (PMC12479014; doi:10.1371/journal.pgen.1011871)
Supplement: Fig S1 — (PDF) [file pgen.1011871.s009.pdf]

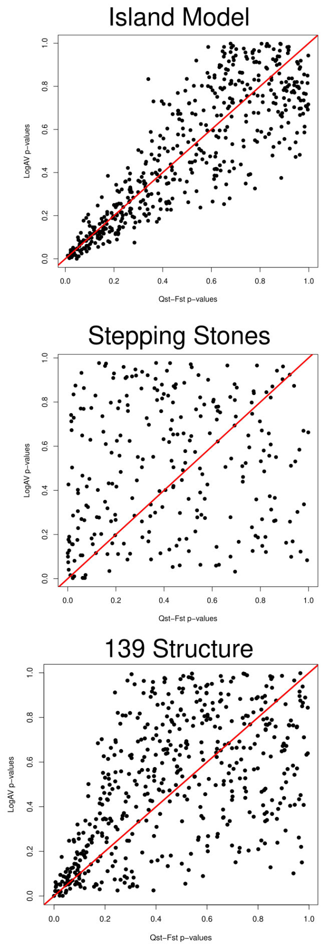

Figure S1: Comparison between distributions of results of the LogAV method and Q_{ST}–F_{ST} over neutraly evolving simulated metapopulations under Island model, Stepping stones, and 139 structure
