# Supplementary material for: A method for identifying local adaptation in structured populations
Source: PLoS Genet. 2025 Sep 23;21(9):e1011871. doi: 10.1371/journal.pgen.1011871 (PMC12479014; doi:10.1371/journal.pgen.1011871)
Supplement: Fig S2 — (PDF) [file pgen.1011871.s010.pdf]

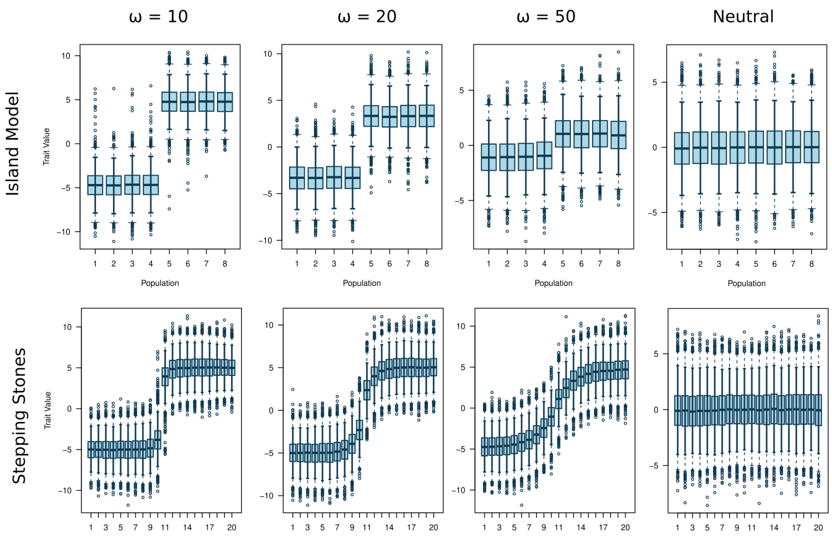

Figure S2: Mean and standard deviation of trait values per subpopulation in different selection regimes for Island Model and Stepping stones.
