# Supplementary material for: A method for identifying local adaptation in structured populations
Source: PLoS Genet. 2025 Sep 23;21(9):e1011871. doi: 10.1371/journal.pgen.1011871 (PMC12479014; doi:10.1371/journal.pgen.1011871)
Supplement: Fig S5 — (PDF) [file pgen.1011871.s013.pdf]

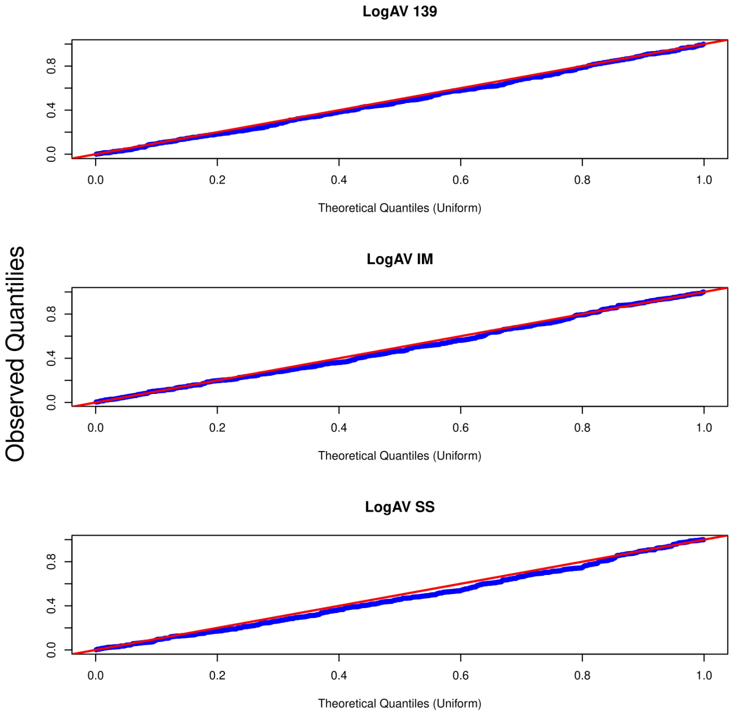

Fig S5. Quantile plots comparing the theoretical expectation under neutrality with the observed distribution for LogAV. We show the comparison for three neutrally evolving population structures.
