# Supplementary material for: A method for identifying local adaptation in structured populations
Source: PLoS Genet. 2025 Sep 23;21(9):e1011871. doi: 10.1371/journal.pgen.1011871 (PMC12479014; doi:10.1371/journal.pgen.1011871)
Supplement: Table S1 — (PDF) [file pgen.1011871.s014.pdf]

Table S1: Summary of simulation parameters for each scenario.

**Scenario**	**Generations**	**Subpop. Size**	**Num. Subpop.**	**Sample per Subpop.**
Neutral IM	500	1000	8	10
Neutral SS low Fst	5000	500	20	10
Neutral SS high Fst	5000	1000	20	10
Neutral 139	900	1000	9	10
IM selection (all)	500	1000	8	10
SS selection (all)	5000	1000	20	10
