# Supplementary material for: A method for identifying local adaptation in structured populations
Source: PLoS Genet. 2025 Sep 23;21(9):e1011871. doi: 10.1371/journal.pgen.1011871 (PMC12479014; doi:10.1371/journal.pgen.1011871)
Supplement: Attachment — Submitted filename: Response_to_reviewers_July8.pdf [file pgen.1011871.s015.pdf]

**Additional Editor Comments :**

Three experts have now reviewed the manuscript, and I have read it as well. All readers are agreed that the proposed approach represents a worthwhile addition to the set of methods available for testing for divergent selection among related natural subpopulations, and all are positive about the fit of the paper with PLoS Genetics. At the same time, the reviewers all suggested additions to the manuscript that would make it clearer or more convincing. In particular, reviewers 1 and 2 ask for additional theoretical and methodological detail, and reviewers 1 and 3 suggest additional simulations that would strengthen the manuscript. Reviewers 2 and 3 both ask the authors to consider whether other schemes for estimating pieces of input to the model that might allow for wider application of the method. In a revised version, I would ask that you comment on these possibilities and/or expand on the necessity for the design proposed.

**Reviewers' comments:**

Reviewer's Responses to Questions

## Reviewer #1

Summary
The authors present a simple, robust method to perform QST-FST analyses under a variety of population and breeding structures. They combine and extend previous work on estimating kinship matrices and applying the animal model to study local adaptation. I think that the developed test quantity of the ratio of the between to within population estimates of the ancestral genetic variance is a good idea that will likely apply to a wide range of scenarios. I do not see any major issues with the paper. The structure of the paper is that of advocating a new methodological approach without providing software to apply the method or testing it on real data. I generally think this is fine, but I think that a bit more should be done to justify the approach and identify conditions under which it will fail.

We thank the reviewer for these positive comments and for the many relevant suggestions on how to improve our manuscript, which we address below. Here we simply want to point out that our revised version now includes a link to the GitHub page [https://github.com/isadoo/MethodsLocalAdaptation_LogAV] where potential users can access the R scripts used for analyzing the simulated data. Additionally, we are in the process of preparing a comprehensive user manual and an associated manuscript that will present an R package implementing the method.

Major comments:
The approach is described in terms of estimating the additive ancestral genetic variance and the provided derivation uses a model of population structure where subpopulations result from one ancestral, panmictic population. However, it is clear that the authors intend for the model to be applicable in scenarios where this is not true. All but one of the simulated models lack any ancestral population. It is therefore strange to see the discussion suggest investigating “to what extent the proposed method is affected by the lack of panmixia in the ancestral population.” The method was tested on three models without any ancestral population at all, and seems to perform quite well, but this is not really discussed or interpreted at all. The authors should do

more to at least discuss this generality. In the absence of any software or empirical examples the main focus must be on the theoretical properties.

We are sorry for the confusion: all the simulations started with one ancestral population, this is now described in Supporting Information section “Quantinemo” (l.765-777).
How should one interpret estimates of the ancestral genetic variance in situations like models 2 and 3? It seems like the ancestral genetic variance may be related to the point at which lineages have been sufficiently shuffled by migration to become exchangeable. Some sort of explanation along these lines would be helpful.

To clarify, we used the default parameters of quantinemo in which allele frequencies for the quantitative trait are drawn in such way to maximize initial genetic diversity at the global level. Such initialization implicitly draws from a panmictic ancestral population. We now add a clarification on the Supporting Information in a section called “Quantinemo”, (l.765-777).

The authors should be more explicit about the variance decomposition leading to equation (8). I was thrown by the seeming possibility for negative values in equation (7) so additional rationale is needed there.

The reviewer is correct in pointing out that elements of the kinship matrix for each subpopulation described by eq. 7 can be negative. We note first that it is so for most genomic estimators of kinship (for instance, the standard estimator also produces negative estimates of kinship, see Goudet et. al., 2018). Additionally, it is not this kinship matrix, but the matrix **M**, that enters variance decomposition, leading to Eq. 8 for the individual component. While **M** also can contain negative values, we found no instances where it is not positive definite (all eigenvalues were always positive) and thus the matrix can be inverted by standard procedures. As for $\boldsymbol{\Theta}^{p}$, we ensure that all its elements are positive by making it relative to the smallest off-diagonal element of the between-population mean allele sharing matrix (eq. 6).

One simple robustness check that could be accomplished with the simulations already performed would be to misassign some individuals to the wrong populations. These may represent recent migrants or incorrect labelling. There is some natural worry with QST methods that populations are correctly defined in the first place. My guess would be that such mislabeling does not lead to false positives since it would inflate within population variance, which the extra between population relatedness would be ignored. This would be good to see explicitly.

We appreciate this suggestion but note that properly addressing this issue would require an extensive set of simulations and would substantially change the focus of our study. For example, exploring the robustness of our method to this problem would require a set of simulations in which we progressively increase the number of individuals that have been mislabeled. But the effect may depend not only on the total number of mislabeled individuals but also on how they are distributed across the studied populations. Its effect may also depend on sample sizes per population, another parameter that would need to be varied.

We believe the problem of mislabeling becomes of concern when delineation of local populations is unclear, in which case it would be preferable to use a method that explicitly considers this type of scenario. An existing method that could be used in this case is presented by Joseph et al. (2019) and is based on the well known principle that hidden population structure can be identified using principal component analysis. In the specific case of Joseph et

al. (2019), the kinship matrix is decomposed into PCs that are used to estimate $V_A$, and an orthogonal set of PCs used to test for selection. We would strongly encourage the use of this approach instead of ours in situations where individuals are not naturally grouped into well defined local populations.

The reviewer also mentions that the suggestion could also apply to recent migrants. However, our simulations consider three different models of population structure and two levels of migration. We, therefore, feel that our simulation study is comprehensive and more detailed than most carried out in the context of studies presenting new statistical methods.

Given the focus on the properties of the allele-sharing estimator of coancestry, it would be good to explicitly see whether using other estimators inflates the rate of false positives.

While we agree it would be interesting to explore other estimators of coancestries, we feel this goes beyond the scope of this article: we provide a working, solid solution to the estimation of coancestries within and between populations. It was shown in Goudet & Weir (2023) that the RAFM estimator of coancestry developed by Karhunen and Ovaskainen (2012) is 1/ less reliable than the allele-sharing estimator and 2/ does not scale to genomic datasets. We are not aware of other estimators of coancestries in this context.

While no software or empirical examples are presented (presumably to be given by future work), it would still be good to know whether the method could be applied to the re-analysis of published data. Driftsel appears to have been applied in at least a handful of published works. Would re-analysis be straightforward?

We thank the reviewer for the point raised. Yes, it should be straightforward to re-analyze data once analyzed using Driftsel. We now include an example of a published dataset that had been previously analyzed using Driftsel in the “Applications” section of our manuscript (l.339-351).

Minor comments:
144-145: FST is not proportional to the average of the diagonal element of Theta. Doubling this average while maintaining between population relatedness would not double FST?

We thank the reviewer for noticing this, and we modified the text, which now reads “$F_{ST}$ is a linear function of the average of the diagonal elements of $\mathbf{\Theta}^p$”

165: I think stabilizing selection on the phenotypic trait, rather than balancing selection, is meant here.

We agree with the reviewer that the phrase ‘balancing selection’ is not the most appropriate to describe the scenario under consideration, for which we apologise. In fact, the two alternative scenarios we consider involve selection acting at two different spatial scales. In the case of local adaptation, there is divergent selection among populations and stabilizing selection within each local population. In other words, local populations evolve towards different optima and, therefore, $V_{A,B} > V_{A,W}$. On the other hand, the alternative scenario, $V_{A,B} < V_{A,W}$, which we now describe as ‘spatially homogeneous, global adaptation’, following Yeaman (2022), corresponds to the situation in which all local populations evolve towards a single global optimum (global stabilizing selection). Thus, the main difference between the two scenarios is the level at which stabilizing selection acts (local versus global). To address this issue, we now provide a more thorough description of the two alternative scenarios that our method can distinguish when the

hypothesis of neutral evolution is rejected (see introduction; lines 78-83). Additionally, and as we mentioned before, we adopt the terminology of Yeaman (2022), i.e., ‘spatially homogeneous, global adaptation’, when referring to the scenario previously described as ‘balancing selection’ (l. 33).

219: It is not sufficient to say that a Bayesian estimation framework is employed without specifying priors and how the model was fit. I don’t see this in the supplement but it should be there.

We apologize for this oversight. We now provide more information on the actual implementation of the Bayesian framework (l.230-233), which is based on the brms package (itself using the STAN library to implement a Hamiltonian Monte Carlo algorithm), using default priors that are very sensible, especially for variance components.

269: AFM was not previously defined.

Thank you for noticing, we have now added a definition next to where it is mentioned (l.71).

## Reviewer #2

In this paper, Isabela et al. developed a new method to estimate the genetic variance between- (Vb) and within-populations (Va) for Qst-Fst comparisons that takes within and between-population relatedness into account. They call this approach LogAV and show that its Type I error is better calibrated than other approaches (e.g. traditional Qst-Fst and DriftSel) for a range of population structures.

The approach makes sense and the authors convincingly show that the errors are well-calibrated. My main comments are related to the lack of sufficient details of the methods and simulations:

I found the details of the inference to be a bit sparse in the main text and supplement. I understand that a Bayesian framework was used to estimate Va and Vb but what were the priors? I assume uniform given their discussion on the equivalence of p-values and posterior probabilities but please make this explicit. What was the range of the prior distribution? Which algorithm was used to sample from the posterior? The code on github suggests MCMC but this and the specific packages used should be explained in more detail in the paper, especially since this is primarily a methods paper.

We apologize for this oversight. See our response to reviewer 1 for more details on the implementation of the Bayesian framework. We used the default priors of brms, which are very sensible for variance components.

Please provide more details on the simulation parameters. 100 causal and 2000 neutral markers were used for the simulations. What were their frequencies? Were they drawn from some distribution? Is there a burn-in period before divergence? How exactly were the genetic values simulated to ensure heritability of 0.8 across all scenarios?
Was coancestry estimated from neutral markers only or were the causal variants included? This matters because the frequency distribution and Fst can differ between neutrally evolving markers and those under selection and it's important to evaluate how that would affect estimation of variance components with LogAV.

Coancestry was estimated from neutral markers only. We acknowledge that in practice it will be difficult to identify neutral markers only (some methods exist, e.g. Pouyet et al, 2018 https://doi.org/10.7554/eLife.36317). However, we believe this won't affect the estimation of coancestries too much: if the trait is oligogenic, few QTLs will show very large $F_{ST}$, but they will be diluted among the many neutral markers; if the trait is polygenic, each QTL's $F_{ST}$ won't differ much from neutral ones (see Le Corre and Kremer 2012 for instance). We mention that on the Supporting Information, section "Data for calculating coancestries" (l. 775-787).

Fig. S2 is supposed to show 100% success of LogAV in detecting selection. The figure is difficult to interpret beyond showing that the p-values under the neutral scenario are uniformly distributed and under selection to be very low. A better, more informative visualization would be an ROC curve.

We thank the reviewer for the suggestion of a better visualization. We now include simulations with smaller strength of stabilising selection, which allows for a better visualization of potential results (Described on Comparison and verification). As for the ROC, we believe it does not fit well with the type of data we are showing, since we are not comparing different methods in the selection scenario, systematically with neutral and selected traits. Nevertheless we show here what a graph as close as possible to a ROC curve would look like:

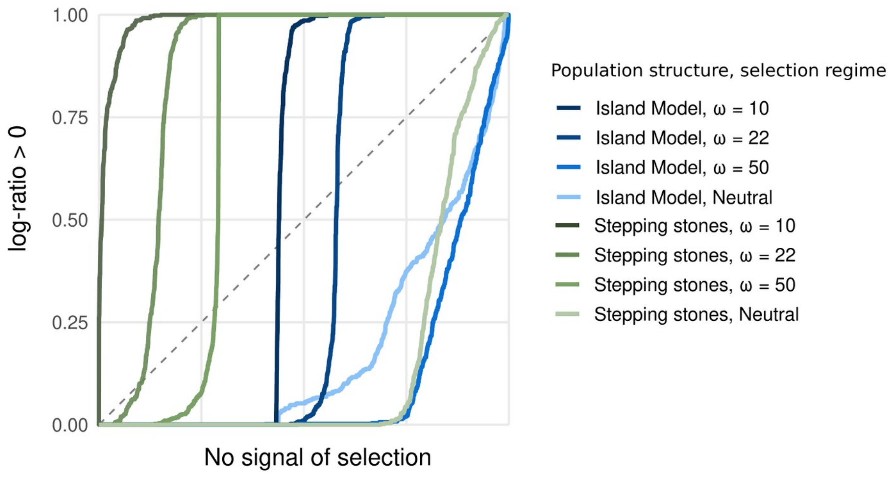


Do you strictly need a design where genotypes from the parents are available? My intuition is that it should be possible to get an estimate of theta^p from the offspring generation by methods such as REAP (Thornton et al.) and PC-RELATE (Conomos et al.) to estimate it.

The reviewer is correct that genotypes for the parents are not strictly necessary, genotypes for any individuals from the parental generation would be suitable to infer $\Theta^p$**.** It might indeed be possible to estimate $\Theta^p$ from the offspring generation, but caution is needed: using all F1 genotypes will lead to inflated $\Theta^p$ values; a subset of independent individuals might work, but needs to be tested. One might also consider reconstructing parental genotypes from their offspring, but we have not explored these possibilities. We have added mention of this issue in

the Discussion (l.501-507).

Given that this is primarily a method paper, these details should be made more explicitly available so we can evaluate the performance of the method under different scenarios. It would also broaden the scope of the paper to describe the performance of the method under a wider range of scenarios considering different strengths of selection and genetic architectures. Without this, the paper comes off as more of a preliminary analysis of a promising method as opposed to a fully realized approach.

Thank you for your suggestion, we now include tests on simulations with other selection scenarios, specifically with smaller strength of stabilising selection for two of the population structures.

Reviewer #3

The manuscript by do O et al explores a new test for selection on quantitative traits by building a linear mixed model and testing for equivalent of the "ancestral population variance" and the within population variance. The test is conceptually similar to Qst-Fst tests, but, as the authors note, expected to be more robust because it can account for more complicated forms of population structure. It also produces something akin to a calibrated p-value distribution, which seems robust to population structure.

I think that the method overall sounds quite reasonable: the authors form two genetic relatedness matrices, one account for the relationship between populations and one accounting for the relationships within each population. They then cleverly use this to build a a statistical test that the within and between population variances are the same. They assess significance using a Bayesian p-value, which is not a concept I was familiar with.

I think that their simulation results, though somewhat sparse, suggest that this method controls false discoveries significantly better than alternative approaches, particularly when population structure deviates from an island model.

While the authors mostly focus on false positive rates, and do a thorough job exploring different scenarios, they only spend a short time (and one supplementary figure) on the power of the test, which they find to be 100% in the scenario they explored. I think that an easy way to make the manuscript have a little more meat would be for the authors to explore a bit more about the power of the method, for example by exploring power across a grid of omega or optimum shift values. It would also be interesting to compare power to the other methods---although given their elevated false positive rate, the authors would need to be somewhat careful about how they frame it.

We thank the reviewer for those suggestions, and we now include four more selection scenarios with smaller strength of stabilising selection.

Another thought that struck me during the reading of the manuscript is that the authors imagine a two step procedure for data collection: first, sampling parents in the field and using them to build the \Theta matrix, and then doing a common garden experiment using the offspring of those samples to measure phenotypes and to measure genotypes to build the M matrix. First of all, it seems like (and perhaps I'm overlooking some aspect of double dipping here) that the

same F1 genotypes could be used to construct M and \Theta, which could potentially be useful in some settings?

We thank the reviewer for this interesting point. As similarly stated above to Reviewer 2, it is possible to use the F1 genotypes to infer the parental genotypes, or even to directly calculate $\boldsymbol{\Theta}^p$. However, also as stated, caution is advised due to potential inflation of $\boldsymbol{\Theta}^p$ values (l.501-507)

Secondly, and maybe more interestingly, what if phenotypes and genotypes are measured exclusively from the parental generation? There are many systems in which a common garden is not feasible, but questions of this sort are still interesting. This framework, based entirely on genetic relatedness matrices, seems like it could in principle be applied using purely parental genotypes and phenotypes. Obviously a complication here is that environmental differences between the parental populations will confound the estimation of V_{A,B}. However, it would be interesting to see how strong of an effect would be necessary to really mess up the test. I'm not suggesting that the authors have to do this, as I think he manuscript is already very interesting, but it could be of interest to many practitioners---and anyway, I have a strong suspicion some might just go ahead and do it!

Thank you for your interesting question. We have added to the Discussion on the matter of measuring phenotypes directly in the field rather than in a common garden (l. 483-494).

Finally I think that the authors test bears some similarity to a few tests proposed elsewhere in the literature that I think the authors should engage with, e.g. Warnefors and Eyre-Walker (2012 and Latrille et al (2024), which if I recall correctly test a very similar hypothesis about equal variances but motivate it from a McDonald-Kreitman-like perspective. To be clear I absolutely do not think the current manuscript is duplicative of the work in these papers, but they are similar enough that it's probably worth discussing.

The two references the reviewer points out are indeed relevant to our work and we now include them in our discussion (l.436-445).

I prefer to sign my reviews. My name is Joshua Schraiber.
