# Supplementary material for: A method for identifying local adaptation in structured populations
Source: PLoS Genet. 2025 Sep 23;21(9):e1011871. doi: 10.1371/journal.pgen.1011871 (PMC12479014; doi:10.1371/journal.pgen.1011871)
Supplement: Attachment — Submitted filename: responses_to_reviewers.pdf [file pgen.1011871.s016.pdf]

Reviewers' comments:

Reviewer's Responses to Questions

Reviewer #1: I thank the authors for their responses to my comments and for the changes made to clarify their analysis and improve the manuscript. While the authors did not investigate some of the robustness issues I brought up in my original review, the power simulations and empirical examples are probably more worthwhile additions. The only necessary additions are code and data to reproduce the empirical results. It would also be good to have power simulations of spatially-homogeneous global adaptation, but this is arguably not as interesting as local adaptation.

Code availability:
The GitHub link provides sufficient code and documentation for the generating and analysis of simulated data. I am very happy to see the empirical example where LogAV is applied to the Stickleback data. I would only add that the scripts and ideally data files used to produce Table 3 should also be added to the repository.
We apologize for the oversight of not updating the GitHub repository. We have now updated the directory and all code and data is fully available there.

Population structure in simulations:
My original confusion with the models of population structure in simulations stemmed from the diagrams in figures 4 and 5 as well as the description in the first paragraph of the "Verification and Comparison" section. No lines are drawn to indicate an ancestral population. In particular, the standard island and stepping stone models of population structure are most often at equilibrium, where the structure persists indefinitely into the past. The authors have pointed out that because of how these simulations are initialized, and the fact that they are not run long enough to reach equilibrium, it is equivalent to having an ancestral population. I don't expect it makes that much difference to the results, it would be good to explicitly state in the caption to Figure 4. I don't see any reason not to and it would help clarify things a bit for readers who may naturally assume that such models are the equilibrium versions. I don't think anything else is necessary here.
Thank you for your suggestion. We have now added to the legend of Figure 4 an explanation about the ancestry.

Selection simulations:
The addition of Figure 6 is an improvement over what was given in the previous version of the manuscript and the addition of simulations of weaker diversifying selection is welcome as well. It would have been nice to have matching power simulations under the spatially-homogeneous global adaptation model, but I understand that studies using the method would be more interested in finding local adaptation.

Re-analysis of stickleback data:
I find the empirical example to be a great addition to the paper. This demonstrates that the method will work on real-world data even though the full software implementation is not yet available. I would only ask the authors to make these scripts available.

Thank you for your comments, we have added the scripts and data to the GitHub repository.

Reviewer #2: I am overall satisfied with the revisions made by the authors and have no further major revisions to suggest. I would recommend one minor clarification:

Regarding the possibility of estimating theta^p and M from the offspring, the authors give two different reasons for cautioning against this: (i) using all F1 genotypes will lead to inflated theta^p values (in response to my comment), and (ii) inflated theta^p values due to the added drift across generations in the common garden experiment (in the Methods section of the main text). Could they elaborate on their reasoning or provide an appropriate citation? If my intuition is correct, if we have allele frequencies from the sub-populations (e.g. from a reference set), we should be able to estimate the drift in the sub-populations, and therefore, theta^p fairly easily. Could the authors clarify?

We did not find where in the main text a sentence reflecting point point (ii) above, but we've added the word ideally to the sentence l 177 below and we elaborate on this in supplementary section "Method testing details", subsection "Data for collecting coancestries", and suggest alternatives ways of estimating theta^p if neutral markers from the parental generation are not available:

"The use of our method **ideally** requires phenotypic data of the offspring generation (which we will call F1) reared in a common environment (i.e.\ ``common garden"), neutral genetic markers from the parental $(\mathscr{P})$ generation, and neutral genetic markers or a pedigree for the F1 **(we discuss alternatives to this ideal setting in Supplementary Text S7)**"

Reviewer #3: The revision by do O et al addresses some of my main concerns. I continue to think the manuscript is a little bit terse, but I think the authors added some meat and improved it by adding some more detailed power analysis and by including an analysis of real data. Though the authors did not do any simulations under a model where phenotypes are only measured "in the wild", I think their discussion of the point is acceptable. Regarding the asks in my original review, I feel satisfied by the authors' response.

Thank you for your comments!

I prefer to sign my reviews. My name is Joshua Schraiber.
